# Supplementary material for: Role of Copeptin and hs-cTnT to Discriminate AHF from Uncomplicated NSTE-ACS with Baseline Elevated hs-cTnT—A Derivation and External Validation Study
Source: Cells. 2023 Mar 31;12(7):1062. doi: 10.3390/cells12071062 (PMC10092967; doi:10.3390/cells12071062)
Supplement: Supplementary file 1 [file cells-12-01062-s001.zip › cells-2229682-supplementary.pdf]

Supplement Material

Figure S1. Consort diagram validation cohort

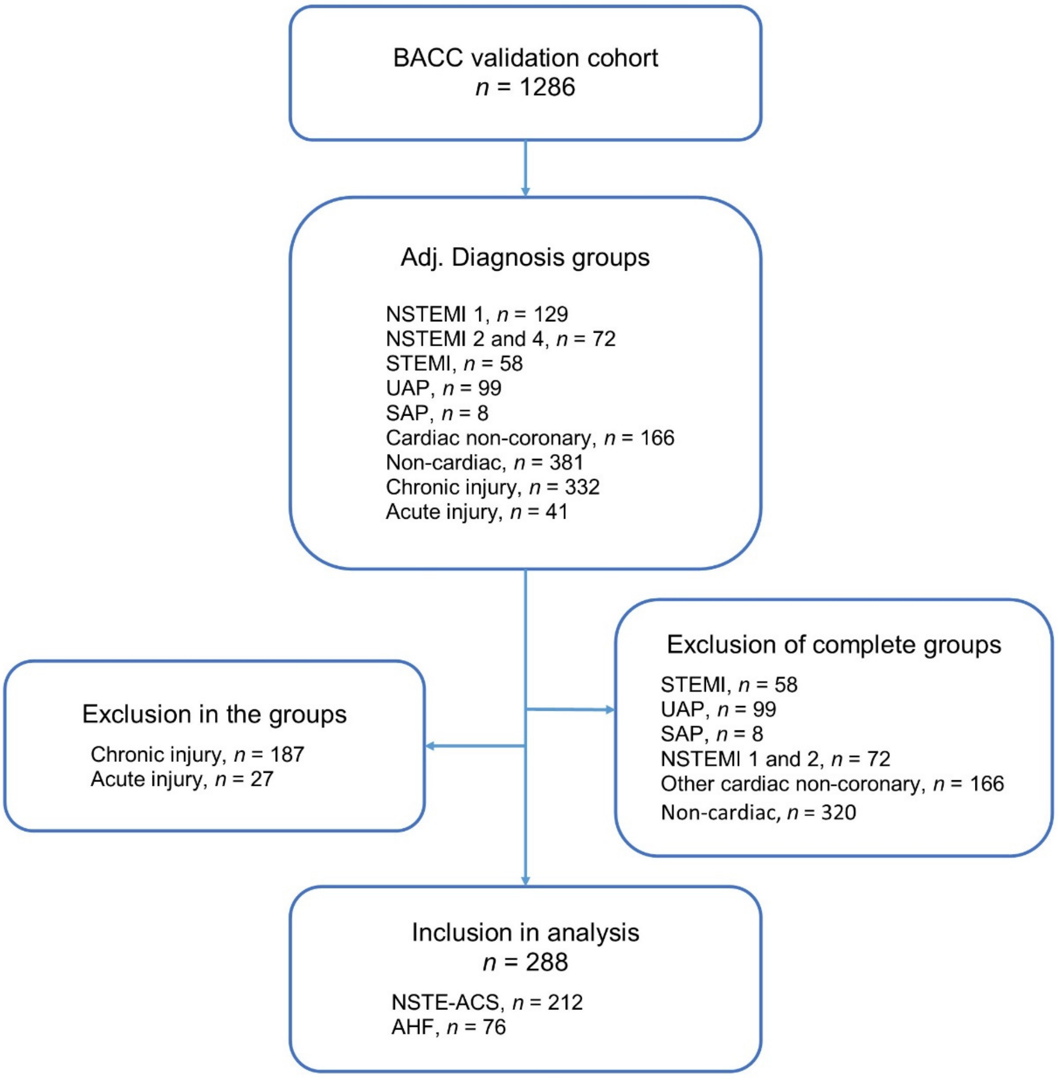

Figure S2. A) ROC Comparison of discriminatory ability of Copeptin, hs-cTnT at 0 h and hs-cTnT at 3 hours at validation cohort. B) ROC Comparison of discriminatory ability of hs-cTnT as maximal concentration and concentration change at validation cohort

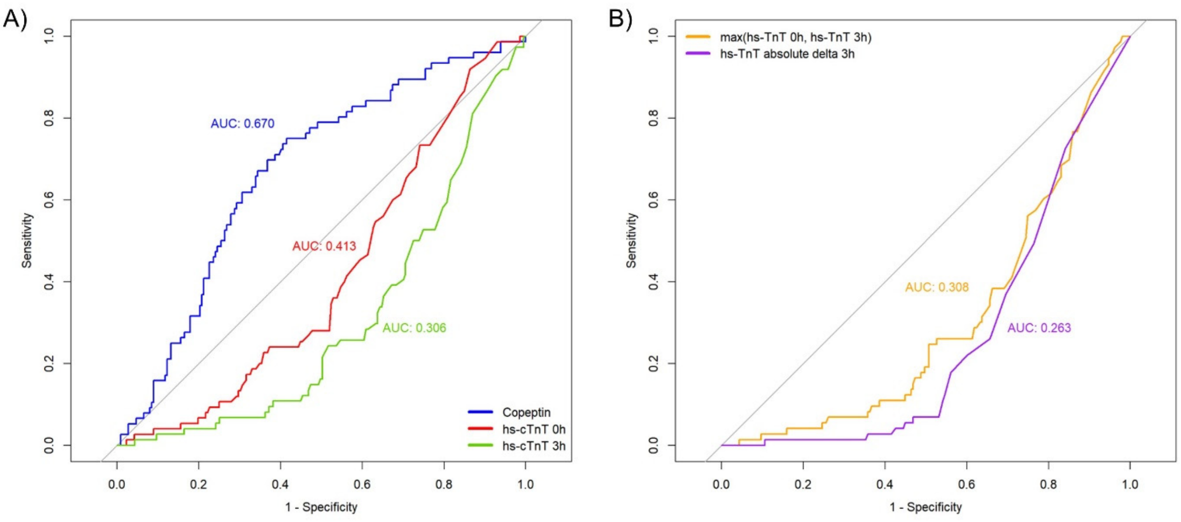

Figure S3A. Kaplan-Meier curves, Copeptin ≤45 vs. >45 pmol/L by all-cause death at 365 days

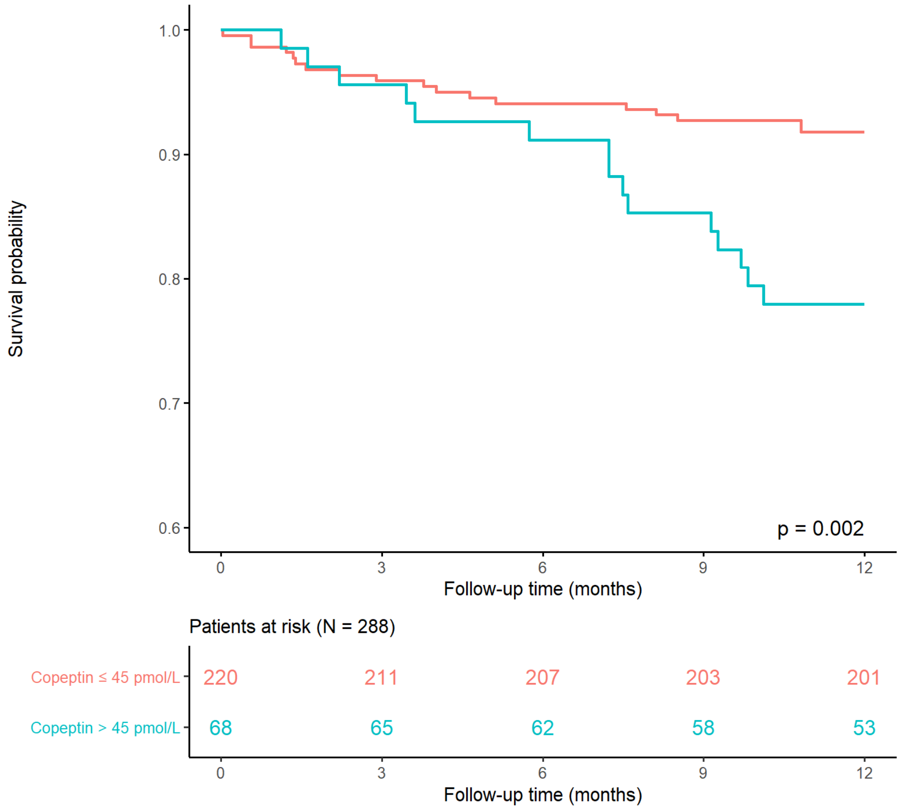

Figure S3B. Kaplan-Meier curves, hs-cTnT at 0h ≤74 vs. >74 ng/L by all-cause death at 365 days

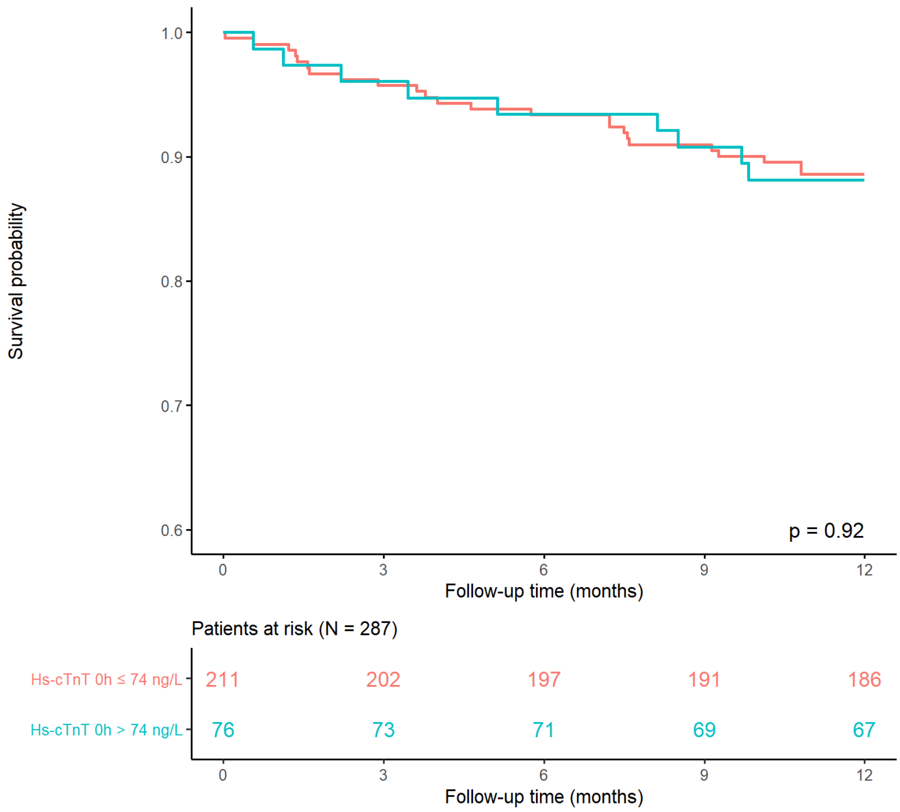

Figure S3C. Kaplan-Meier curves, hs-cTnT at 3h ≤78 vs. >78 ng/L by all-cause death at 365 days

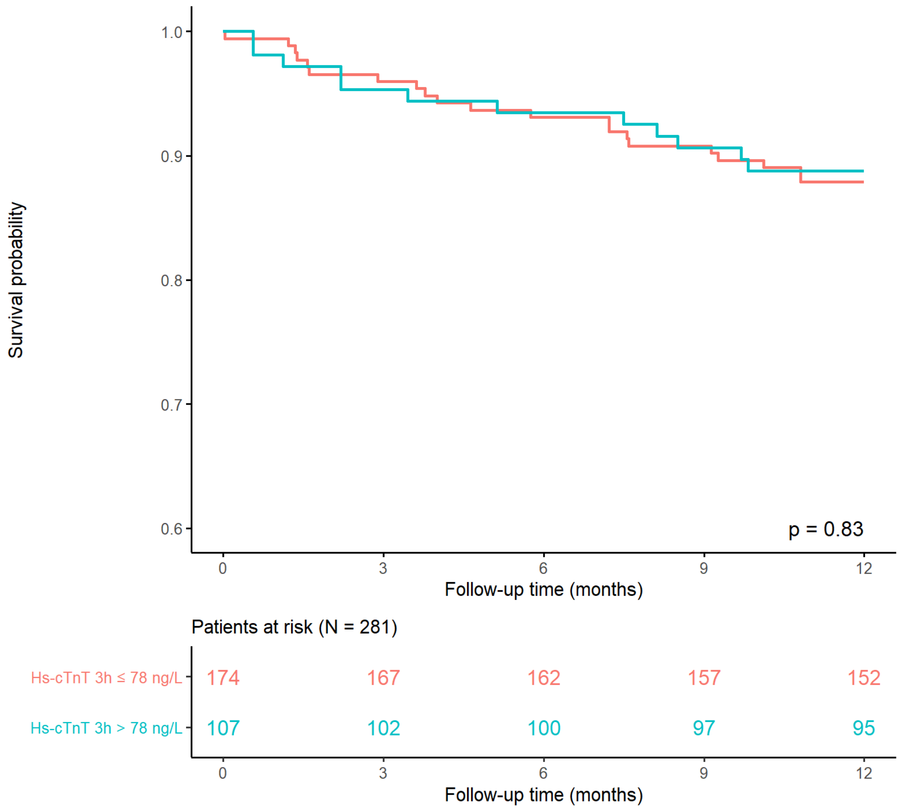

Table S1. Baseline characteristics and laboratory values in validation cohort

	All Patients (n=288)	Acute HF (n=76)	NSTE-ACS (n=212)	p-value
Age	72.0 (60.8-78.0)	73.0 (65.8-80.2)	71.0 (60.0-78.0)	0.14
Age > 75 years	101 (35.1)	31 (40.8)	70 (33.0)	0.28
Male (%)	201 (69.8)	53 (69.7)	148 (69.8)	1.00
eGFR (ml/min/1.73m²)	64.4 (46.2-80.8)	48.6 (33.5-70.1)	69.7 (51.1-85.1)	<0.001
eGFR < 60 ml/min/1.73m²	130 (45.5)	51 (67.1)	79 (37.6)	<0.001
eGFR < 30 ml/min/1.73m²	27 (9.4)	14 (18.4)	13 (6.2)	0.0038
**CV Risk**				
Hypertension, No. (%)	245 (85.4)	70 (92.1)	175 (82.9)	0.080
Diabetes mellitus, No. (%)	73 (25.4)	23 (30.7)	50 (23.6)	0.29
Hypercholesterolemia, No. (%)	180 (62.5)	48 (63.2)	132 (62.3)	1.00
Chief complaint				
Chest Pain 48h before admission, No. (%)	226 (78.5)	44 (57.9)	182 (85.8)	<0.001
Dyspnea, No. (%)	123 (44.9)	52 (69.3)	71 (35.7)	<0.001
**Medical history**				
History of AMI No. (%)	91 (31.7)	21 (27.6)	70 (33.2)	0.46
History of CAD/Bypass/PCI No. (%)	177 (61.5)	43 (56.6)	134 (63.2)	0.38
History of PAD No. (%)	30 (10.4)	8 (10.5)	22 (10.4)	1.00
History of HF No. (%)	100 (34.7)	59 (77.6)	41 (19.3)	<0.001
GRACE score	125.0 (103.0-148.2)	149.0 (131.8-166.0)	117.5 (95.8-136.0)	<0.001
**Laboratory**				
Copeptin (pmol/L)	13.4 (5.5-41.5)	27.2 (12.8-59.9)	9.6 (4.9-28.0)	<0.001
Hs-cTnT 0 h (ng/L)	32.0 (18.0-87.0)	27.0 (17.0-42.0)	38.0 (18.0-120.5)	0.025
Hs-cTnT 3 h (ng/L)	48.0 (22.0-153.0)	26.0 (17.0-50.2)	67.0 (24.5-201.0)	<0.001
Hs-cTnT delta	5.5 (2.0-32.0)	2.0 (1.0-5.0)	10.0 (3.0-55.0)	<0.001
Maximum hs-cTnT	41.0 (21.0-106.0)	27.0 (18.0-52.0)	50.0 (24.0-156.2)	<0.001

Table S2. Cox regression models in validation cohort

	Univariate Model		Multivariate Model 1*		Multivariate Model 2**	
	**HR (95% CI)**	**p-value**	**HR (95% CI)**	**p-value**	**HR (95% CI)**	**p-value**
**Copeptin (log)**	1.54(1.21-1.96)	<0.001	1.37 (1.06-1.77)	0.017	1.37 (1.06-1.78)	0.017
**Hs-cTnT 0h (log)**	1.19(0.92-1.53)	0.19			1.20 (0.92-1.58)	0.180
**Hs-cTnT 3h (log)**	1.09(0.85-1.39)	0.52				
**Age**	1.07(1.04-1.11)	<0.001	1.06 (1.02-1.10)	0.002	1.06 (1.02-1.10)	0.003
**Male**	0.64(0.32-1.29)	0.21	0.90 (0.43-1.87)	0.78	0.89 (0.43-1.86)	0.750

***Model 1 was adjusted for copeptin, age and sex. **Model 2 was adjusted for copeptin, hs-cTnT, age and sex.**
